# Oregon's Behavioral Health Initiative for Older Adults and People With Disabilities at 5 Years: Where to From Here?

**DOI:** 10.1093/geroni/igaa057.113

**Published:** 2020-12-16

**Authors:** Walter Dawson, Allyson Stodola, Serena Hasworth, Jason Kyler-Yano, Jaclyn Winfree, Linda Dreyer

**Affiliations:** 1 Oregon Health & Science University, Portland, Oregon, United States; 2 Portland State University, Portland, Oregon, United States

## Abstract

Oregon’s Behavioral Health Initiative for Older Adults and People with Disabilities is entering its fifth year. This novel state-level Initiative seeks to better coordinate services and resources for older adults and people with disabilities who have behavioral health needs by assigning a Behavioral Health Specialist (BHS) for every 60,000 adults 65 + and embedding them within local service agencies around Oregon. BHS primary job functions include improving coordination and collaboration between local service agencies, providing complex case consultations (CCC), and delivering workforce development training and community education. Five years of data from Portland State University’s Institute on Aging’s ongoing evaluation of the Initiative suggests significant impact in terms of workforce development trainings, community education, and new community partnerships. Data are collected from BHS and Initiative stakeholders (e.g., aging services agencies). Data collection tools include quarterly reports from the BHS, including a CCC reporting instrument; semi-structured interviews with stakeholders assessing Initiative involvement; and an electronic post-training survey (and two-month follow-up survey) for stakeholders attending BHS trainings. After five years, the evaluation appears to show the Initiative has delivered an abundance of innovative collaborations, workforce trainings, and educational opportunities aimed at better supporting the behavioral health of older Oregonians. It also highlights several persistent systemic barriers including a need for additional public funding of behavioral health, the challenges of accessing Medicare for behavioral health, and siloed agencies and organizations. Future evaluative efforts could explore adding outcomes-based assessments of the Initiative, including local-level quality improvement projects initiated by BHS within their communities.

